# Force and the spindle: Mechanical cues in mitotic spindle orientation

**DOI:** 10.1016/j.semcdb.2014.07.008

**Published:** 2014-10

**Authors:** Alexander Nestor-Bergmann, Georgina Goddard, Sarah Woolner

**Affiliations:** Faculty of Life Sciences, University of Manchester, Oxford Road, Manchester M13 9PT, United Kingdom

## Abstract

The mechanical environment of a cell has a profound effect on its behaviour, from dictating cell shape to driving the transcription of specific genes. Recent studies have demonstrated that mechanical forces play a key role in orienting the mitotic spindle, and therefore cell division, in both single cells and tissues. Whilst the molecular machinery that mediates the link between external force and the mitotic spindle remains largely unknown, it is becoming increasingly clear that this is a widely used mechanism which could prove vital for coordinating cell division orientation across tissues in a variety of contexts.

## 1 Introduction

The orientation of the mitotic spindle must be carefully controlled in both embryonic and adult tissues in order to regulate cell fate, generate tissue shape and maintain tissue architecture. In embryos, defective orientation leads to failures in morphogenesis and organogenesis [1], [2], while in adults it is linked to cancer [3], [4]. Most investigations of spindle orientation have concentrated on regulation by intrinsic cellular machinery and its upstream regulation by cell polarity (reviewed in [5], [6]). However, recent work has suggested that extrinsic mechanical cues can also direct spindle orientation. A link between mechanical cues and the mitotic spindle has important implications for controlling cell division orientation in tissues. This is especially true in contexts where the mechanical tissue environment undergoes rapid changes, such as during morphogenesis, or is chronically altered by disease, as occurs during tumorigenesis [7], [8].

Cells in a tissue experience a variety of mechanical forces, which include tensile (stretching), compressive (pushing) and shearing (acting in opposing directions) forces. Work over many years has shown that cells are able to sense and respond to these forces through a series of complex processes known collectively as mechanosensing and mechanotransduction (reviewed in [9], [10]). The downstream consequences of mechanical stimuli affect a wide range of cellular behaviours, including cell shape, cell proliferation, gene expression, as well as cell division orientation [11], [12], [13], [14], [15]. The idea that forces play vital roles in cells and tissues is not especially new – biologists have been studying them since the 19th century – but recent developments in the methods used to study forces in biological systems have allowed important new advances to be made. This has certainly been the case in the study of cell division orientation, where the use of biophysical tools such as laser cutting devices, micropatterned substrates and cell stretching apparatus have all been coupled with high resolution live cell imaging to give new insights into how mechanical force influences division orientation. Moreover, the increasing crossover between biology, maths and physics has been vital, both to the development of these biophysical tools and also to the interpretation of their results, allowing us to build predictive mathematical models that can be tested experimentally.

In this review we discuss recent findings from both cultured cells and *in vivo* tissues, which have demonstrated a role for mechanical force in mitotic spindle orientation. We then go on to discuss why a link between external force and cell division orientation might be useful in tissues. Finally, we examine how a link between mechanical cues and the spindle might be mediated and discuss whether the contribution of cell geometry can ever be differentiated from a more direct role for force in spindle orientation.

## 2 Mitotic spindles align with mechanical forces in single cells

**Fig. 1:**
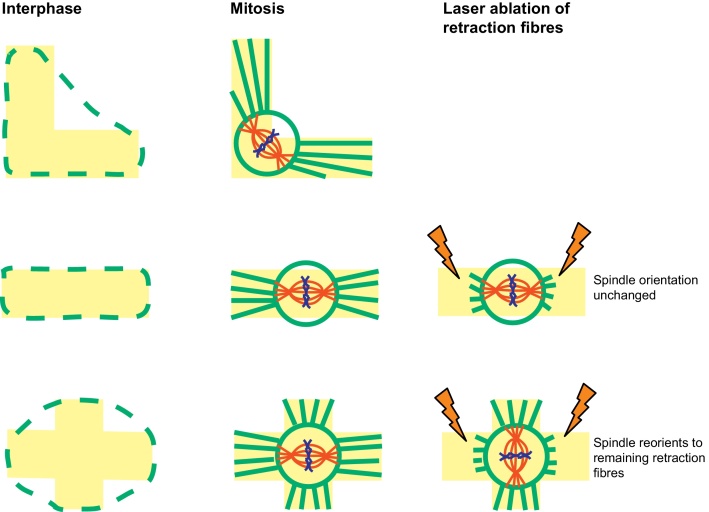
Mitotic spindle orientation in cells grown on micropatterned substrates. Fibronectin (yellow) is micro-contact printed onto glass coverslips and cells are plated onto the adhesive patterns. In interphase cell shape follows the micropatterns (left, green dashed line), in mitosis cells round up but retraction fibres stretch over the micropattern (centre, green lines). The alignment of the mitotic spindle and, therefore, cell division, is dictated by the organization of retraction fibres. If retraction fibres on an asymmetric cross shape are laser ablated the spindle reorients. (For interpretation of the references to color in this figure legend, the reader is referred to the web version of this article.)

*Source*: Based on data from [14], [17].

The first clues that mechanical cues might be involved in orienting the mitotic spindle came from a series of elegant experiments using single cells grown on micropatterned adhesive substrates [14], [16], [17]. In these experiments, fibronectin, a key component of the extracellular matrix, is micro-contact printed onto glass coverslips to generate a variety of adhesive shapes. When interphase HeLa cells are plated onto these adhesive micropatterns they adapt to these shapes, such that a HeLa cell plated on a bar-shaped adhesive pattern will adopt a rectangular shape, whilst a cell plated on an “L” shaped pattern adopts a triangular shape [17] (see Fig. 1). Crucially, when cells subsequently enter mitosis, the mitotic spindle aligns with the cell shape that was determined in interphase by the adhesive pattern, generally aligning with the long axis of this shape [17]. These simple observations indicated that the adhesive contacts that a cell makes with its external environment are key in determining the orientation of the mitotic spindle and, therefore, in determining the orientation of cell division.

The adhesive environment of a cell is known to exert mechanical forces on the cell [18]. Therefore, the finding that this adhesive environment determined spindle orientation suggested that external mechanical forces may control spindle orientation more directly. To test this, Fink et al. applied a unidirectional stretch to cultured HeLa cells and found that spindles rotated towards the applied stretch, indicating that spindles do align with external forces [14]. Similarly, cultured keratinocytes will also align their spindles to an applied unidirectional stretch [19]. In addition to aligning with tensile (stretching) forces, spindles in single cells have also been shown to be directed by shear forces. Shearing forces occur when one part of a cell is pushed in the opposite direction to another part of the cell; the most widely studied examples of cells experiencing these forces are in blood vessels, where blood flow exerts a shear stress on endothelial cells lining the blood vessel [20]. When RPE1 and MC3T3 cells were grown under conditions of shear strain, mitotic spindles were found to orient in a direction perpendicular to the applied shear forces [21]. It is interesting to note that this orientation is opposite to that seen with tensile forces, where the spindle aligns along the axis of stress. A clue as to the reason for this difference may come from cell shape, as tensile forces elongate a cell along the axis of stress whereas cells under shearing forces elongate perpendicular to the applied stress. We will return to the contribution of cell shape in mitotic spindle orientation later in this review, in the section entitled “Is cell shape the key determinant of spindle orientation to external force?”.

## 3 Retraction fibres provide a map of adhesion to which the spindle orients

Many cells round up during mitosis, yet their divisions appear to align with the adhesive environment and cell shape that they were exposed to during interphase. This begs the question of how force and shape information is transmitted to the mitotic spindle and whether this is set in interphase or continually monitored by the spindle during mitosis. In cultured cells retraction fibres appear to play a key role. Retraction fibres are membrane tubes filled with actin filaments – they act as anchors attaching the mitotic cell to the extracellular matrix. In experiments using fibronectin micropatterns, the organization of retraction fibres mirrors the adhesive pattern and the subsequent orientation of the spindle. So, for example, on an L-shaped micropattern the retraction fibres extend from the rounded cell to each end of the “L” (see Fig. 1) [17].

To demonstrate a functional role for retraction fibres in spindle orientation, Fink and colleagues combined adhesive micropatterns with laser ablation to remove particular retraction fibres. Specifically, they grew HeLa cells on either bar or asymmetric cross-shaped patterns and allowed cells to enter mitosis and the spindle to align with the adhesive pattern (with the spindle aligning with the long axis of the adhesive shape in each case). Once the spindles were aligned, the retraction fibres adjacent to each end of the spindle were cut by laser ablation. On the cross-shaped pattern, spindles rotated and aligned with the perpendicular axis dictated by the remaining retraction fibres. In contrast, on the bar-shaped pattern, where no retraction fibres remained, no significant spindle reorientation was observed [14]. Crucially, these experiments demonstrate that retraction fibres are not merely the remnants of interphase cell polarity but are actively involved in spindle orientation. Moreover, the finding that spindles reorient once retraction fibres are cut demonstrates that the spindle is not simply following shape/force cues laid down during interphase but is instead continuing to monitor the adhesive environment and adjusting its orientation accordingly.

## 4 Strong forces are exerted on the mitotic cell body by retraction fibres

So, how do retraction fibres transmit information about the adhesive pattern to the mitotic cell? One possibility is that retraction fibres themselves are exerting force on the cell that is then “read” by the spindle, such that stretching a cell using an externally applied unidirectional force would be analogous to plating the cell on a bar-shaped adhesive pattern. The high tensile forces exerted by retraction fibres have been observed by studying both the cell substrate and the cell itself. If mitotic cells are plated on a thin silicone-rubber layer, the forces translated through the retraction fibres are strong enough to leave wrinkles in the silicone-rubber [22]. Moreover, when retraction fibres are laser cut, the cell body is observed to undergo a mechanical recoil, indicating that the fibres are under high tension [14]. Further studies using optical tweezers to measure the pulling forces exerted by retraction fibres have demonstrated that each fibre carries a load of 245 piconewtons, which translates into a force of about 7 nanonewtons on each side of a cell grown on a bar-shaped pattern [14]. This force, if applied artificially, would result in a significant deformation of the cell. Therefore, whether cells are plated on an adhesive micropattern or subjected to an externally applied force, it appears likely that the same mechanisms are being activated, which allow the cell to align the spindle to the adhesive pattern/force field.

## 5 Does mechanical force orient cell division in tissues?

It is clear from the work detailed above that single cells in culture are able to orient their mitotic spindles according to mechanical forces, whether that be a force induced by an adhesive pattern [14], [16], [17] or by an externally applied strain [14], [19], [21]. However, it has only been in the last couple of years that conclusive evidence has emerged to demonstrate that this is also the case for cells in the tissues of living organisms. Specifically, work in zebrafish [15] and the fruit fly *Drosophila melanogaster* [23], [24], [25] has indicated that mechanical force is an important cue for mitotic spindle orientation in tissues.

During embryogenesis and tissue morphogenesis it is imperative that mitotic spindle orientation, and thus cell division, is carefully controlled in order to regulate the shape, architecture and composition of the tissue [1], [2]. During gastrulation in the zebrafish embryo the epithelial tissue of the animal pole – the enveloping cell layer (EVL) – spreads to cover the entire spherical yolk cell in a process called epiboly [26]. At this stage, the cells of the EVL are also undergoing rapid cell divisions. These divisions are oriented within the plane of the epithelial tissue but, crucially, are also oriented along the animal-vegetal axis – aligning with the direction of tissue spreading [15], [27]. The origin of the control of the animal-vegetal orientation was unknown but one possibility was that mitotic spindles were aligning with a tissue-wide pattern of mechanical tension. To assess whether mechanical force could be a cue for cell division in this tissue, the distribution of tension was assessed to see if it matched the pattern of cell division orientation. A relatively common approach used to measure tension in a tissue is laser ablation: small cuts in the tissue are made by a laser and then the direction and velocity of cell recoil at these sites is measured and used to model tissue tensions [7]. In this way, Campinho and colleagues assessed tension in the EVL, making laser cuts of apical cell cortices in lines parallel and perpendicular to the EVL margin. Measurements of the cell recoil velocity at these cuts revealed an anisotropic (*i.e.* different magnitude in different directions) tension, with greater tension along the animal-vegetal axis than the circumferential axis [15]. Therefore a correlation could be seen between the orientation of divisions along the animal-vegetal axis and the pattern of tension anisotropy. To test whether tension could indeed direct spindle orientation in the epithelium during epiboly, Campinho and colleagues induced a new axis of tension by making small laser wounds in the epithelium. Upon creating two wounds either side of a cell in mitosis, they found that it was possible to switch the orientation of the spindle, causing it to align with the induced axis of tension [15]. Thus, in the zebrafish embryo, spindle orientation is very much influenced by the pattern of tension across the tissue.

Cell division orientation appears to be similarly regulated by a global pattern of mechanical forces in the epithelium of the *Drosophila* wing imaginal disc [23], [24], [25], [28]. The wing disc is an epithelial tissue that undergoes rapid growth – increasing size by over a thousand-fold in five days – and during this growth, cell proliferation and epithelial morphogenesis must be coordinated in order to form a perfect adult wing [1]. Cell divisions in the wing disc occur in the plane of the epithelium but are also oriented in a stereotypical manner within this plane. In the centre of the disc divisions occur along the proximal-distal axis, whilst towards the periphery of the disc the orientation of divisions rotates 90° to give a circumferential pattern [1], [24], [25].

To investigate how these patterns of divisions might be linked to tissue mechanics in the disc a number of approaches have been used to map the patterns of force across the disc epithelium [23], [25]. Analysing changes in cell geometry both in space (across the disc) and time (as the disc grows during development) allowed predictions about the forces acting on cells to be made. These morphometric maps show a concentric pattern of forces across the disc which increases from the centre outwards, with cells at the periphery of the disc experiencing higher levels of stretch than those at the centre [23], [25]. This global pattern of force anisotropy develops as the disc grows. Indeed, computational modelling suggests that the anisotropic distribution of tension could actually be generated by differential proliferation across the disc, with high levels of proliferation in the distal regions inducing the circumferential tension on proximal cells [23]. Laser ablation experiments confirmed the existence of these force anisotropies across the disc by demonstrating that tension at proximal/distal cell junctions increases towards the periphery of the wing disc [25]. So, how do these patterns of forces correlate with the orientation of divisions? The clearest role for mechanical force in cell division orientation is at the periphery of the disc, where cells are experiencing the highest levels of circumferential stretch. In this peripheral region, cell divisions closely match the global pattern of tension revealed by morphometry and laser ablation [23], [25]. In contrast, at the centre of the disc the global pattern of tissue tension appears less important. Instead, the spindles in these cells align with an internally produced tension anisotropy that is generated in each cell by the polarized localization of the atypical myosin, Dachs [23], [24]. We will return to the mechanisms used by the cell to read these internal and external anisotropies below.

## 6 Why might it be useful for cells to orient according to mechanical force?

Orienting the mitotic spindle according to mechanical cues could be a very useful means for globally coordinating cell division across a tissue. This is particularly so in a developing tissue, where rapid proliferation coincides with tissue shaping (morphogenesis). Evidence is growing to show that the link between mechanical force and spindle orientation is vital for coordinating cell division during morphogenesis and for equalizing tissue tension across developing tissues. We will consider these two roles in turn.

**Fig. 2:**
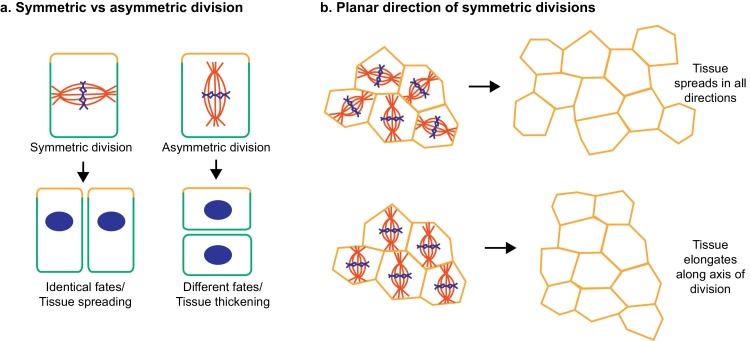
Oriented divisions in epithelia. (a). In polarized epithelial cells, the mitotic spindle can orient to produce symmetric or asymmetric divisions. In symmetric divisions the spindle aligns parallel to the plane of the epithelium, whereas in asymmetric divisions the spindle orients perpendicular. (b). In the case of symmetric divisions a further level of orientation control operates to determine the planar direction of division. This orientation can help determine how a tissue expands – if spindles align in the same direction the tissue will elongate along that axis.

The orientation of cell division can be controlled at a number of levels in a developing tissue. In polarized cells, such as epithelial cells, there is the choice between symmetric and asymmetric divisions (reviewed in [29]). Most cells in an epithelium undergo symmetric divisions, where the spindle aligns parallel to the plane of the epithelium. These divisions are thought to help maintain the organization of the tissue, while also driving tissue expansion [2], [30], [31]. The direction of this tissue expansion can be regulated by another level of cell division orientation: the planar direction of division. If divisions across a tissue are lined up in the same planar direction this can help elongate a tissue along the axis of division. In contrast, if cells in a tissue divide in all planar directions this may help to spread a tissue in all directions (see Fig. 2). Whilst mechanical cues may be involved in both the choice between asymmetric and symmetric divisions and in the planar direction of divisions, here we will concentrate on the latter as, to date, it is only in these divisions that a clear role for mechanical force has been revealed.

The planar direction of cell division has been shown to be important for tissue morphogenesis in a number of different systems. For example, in the *Drosophila* embryo during germband elongation, cell divisions occur in the direction of tissue elongation and are required for this morphogenetic movement to occur properly [31]. Similarly, in *Drosophila* wing imaginal discs, planar cell divisions are oriented in the direction of tissue elongation and a loss of this orientation ultimately leads to misshapen wings in the adult fly [1], [24]. Furthermore, stereotypical planar divisions have been shown to be important during the formation of the neural tube in both zebrafish and *Xenopus* embryos [2], [32], [33], [34].

The orientation of planar cell division can be controlled by the planar cell polarity (PCP) pathway [2], [35], [36]. However, somewhat intriguingly, this has been ruled out in a number of the cases described above. For example, the stereotyped planar divisions seen during germband elongation in the fly embryo and during neural tube closure in the *Xenopus* embryo have been shown to occur normally when the PCP pathway is disrupted [31], [34]. This begs the question, what is controlling these planar cell divisions if the PCP pathway is not? This is still largely unknown, but a strong possibility is that mechanical cues in the tissue may play a role. As described in the section above, planar division orientation in both the zebrafish embryo during gastrulation and in the developing *Drosophila* wing imaginal disc have been shown to be regulated by tissue tension [15], [23], [25]. Although these are the only two examples of mechanical force directing cell division orientation during tissue morphogenesis it seems likely that future work will provide more. Therefore, the ability to link tissue mechanics with mitotic spindle orientation could prove to be a highly conserved mechanism used to coordinate cell division with tissue morphogenesis.

A second suggested role for the control of cell division orientation by mechanical cues is to regulate tension across a tissue. During the growth of a tissue, tension can become highly anisotropic as a result of localized cell proliferation or tissue shaping events [9], [23]. Regulated cell division orientation has been proposed to locally relieve tension and homogenize global stress [37]. In keeping with this idea, Campinho et al. found that there is a reduction in tension following oriented cell division in the zebrafish embryo, which assists in tissue spreading during gastrulation [15]. A similar dissipation of stress by oriented cell division was also observed in the developing *Drosophila* wing disc [25]. In the wing disc it appears that the anisotropic tension is itself caused by differential cell proliferation across the disc [23]. Interestingly, the dissipation of this stress by oriented division is not sufficient to completely alleviate stress within the tissue, and Legoff et al. suggest that a degree of tension may be required to stimulate regions of growth within the tissue and help to generate tissue shape [25]. It is therefore becoming clear that in a developing tissue there is a complex set of relationships linking cell proliferation, tissue tension, oriented division and tissue shape – with each feeding back on the other – of which we have much more to learn.

## 7 Is cell shape the key determinant of spindle orientation to external force?

While the evidence is building that mechanical force is an important determinant of mitotic spindle orientation in both single cells and tissues, the cellular mechanisms controlling this remain largely unknown. One possibility is that cell geometry is intimately involved. Ever since microscopes first allowed biologists to watch cells undergoing mitosis it has been observed that the division plane of a cell is intimately linked to the cell's shape. In 1884, Oscar Hertwig proposed a principle by which cell shape determines cell division axis:

The axis of the spindle lies in the longest axis of the protoplasmic mass, and division therefore tends to cut this axis transversally. [38]

Hertwig's rule, in which the spindle aligns with the longest axis of a dividing cell, remains the most enduring and widely accepted guide for predicting the cell division plane. Since mechanical forces invariably cause a change in cell shape, it is crucial to consider the mechanisms by which cell geometry influences cell division plane.

More recent studies of cell division have built on Hertwig's legacy and further demonstrated that cell geometry is a key determinant of the division plane, with the spindle generally, although not always, aligning with the longest axis of the cell [28], [39], [40]. A beautiful example of such work was carried out by Minc and colleagues using sea urchin eggs and micro-fabricated chambers. The single-celled sea urchin zygotes were pushed into chambers of various shapes, causing them to adopt the chamber geometry, and their subsequent cell divisions were analyzed [39]. Divisions generally followed the long axis of the chamber shape, with some important exceptions: for example, cells in rectangular chambers did not divide along the longest axis, which would be the diagonal axis, but instead divided along the longest axis of symmetry. This work allowed Hertwig's rule to be refined and suggested a model of spindle positioning where the geometry of the cell is read by the length of astral microtubules emanating from the two centrosomes of the spindle – with longer microtubules predicted to generate greater forces to rotate the spindle into position [39].

Cell geometry appears to be particularly important in determining the position of the cell division plane in very early embryos [41], [42], [43], [44], [45], indeed Hertwig developed his theory by observing the initial divisions of echinoderm and frog embryos [40]. However, later in development, especially once cells develop polarity, cell geometry appears to become less important. For example, columnar epithelial cells undergoing symmetric divisions routinely divide against the long axis of the cell, orienting their spindles parallel to the plane of the epithelium when the long axis is perpendicular to this plane (Fig. 2). The control of this division orientation is thought to be dictated by the presence of adherens junctions and cytoskeletal polarity [30], [46], [47]. However, it is important to note that even once a cell is dividing against the long axis, geometry in the dividing axis may still be important. If we return to the example of a symmetrically dividing epithelial cell, while it is dividing against the long axis of the cell, the geometry of the cell in the plane of the epithelium could still be important for determining the planar direction of division (Fig. 2) [15], [34].

## 8 Separating the contributions of cell shape and mechanical force in spindle orientation

It is clear that in many cells, shape plays an important role in determining the orientation of division. Since mechanical forces often change cell shape it is difficult to conclude whether it is simply the change in cell geometry that influences spindle orientation or whether there are additional shape-independent force sensing mechanisms. This conundrum is most easily visualized in the case of tensile (stretching) forces, where the cell divides along the axis of stress but also elongates along that same axis. Therefore, an important question arises: is it possible to separate the contributions of cell geometry and mechanical force in spindle orientation? This is still very much an area of ongoing research but one experiment using micropatterning and applied force has suggested that it is possible to separate shape and force. Fink and colleagues grew cells on elliptical patterns and then applied a unidirectional stretch to produce a perfectly circular ring after stretch [14]. Unstretched cells on the ellipse shape divided according to the long axis of the ellipse, as would be expected. However, when cells grown on ellipses were stretched into rings during mitosis, the spindles turned to align with the axis of stretch, even though the now circular micropattern provided no longest axis. This is in contrast to cells grown on an unstretched circular micropattern, which showed random spindle orientation [14]. Together these results suggest that cells must have a shape independent mechanism of orienting their spindles according to mechanical force.

## 9 The role of actin and myosins in orienting the spindle according to force

Irrespective of the extent to which we believe cell shape is involved in orienting spindles according to force, our understanding of the molecular mechanisms underlying these processes remains very poor. To date, work in both single cells and tissues has pointed to an important role for actin and actin-based motors (myosins) in linking the spindle to external forces. The role of actin in spindle orientation is reviewed in detail in this issue by Lancaster and Baum, so here we will summarize the work specific to orienting the spindle according to mechanical force.

In single cells grown on micropatterned substrates, live imaging has revealed the presence of polarized subcortical actin filaments that mirror the arrangement of retraction fibres [14]. Such dynamic subcortical actin had been observed previously during mitosis in both cultured cells and embryos, although its function was unclear [48], [49]. Fink et al. proposed that these actin structures may provide the link between external force and the spindle: assembling at the site of retraction fibres and recruiting microtubule motors that then pull astral microtubules into position to orient the spindle [14]. This would be similar to the standard model for spindle positioning whereby astral microtubules are anchored at the actin-rich cortex, which provides a platform for the binding of microtubule motors (reviewed in [6], [50]). The major difference being that the actin is now subcortical and sensitive to changes in mechanical force. There are a number of pieces of evidence that support the idea that the subcortical actin plays this role. First, it is in the right place at the right time: the subcortical actin is highly dynamic and localizes close to retraction fibres during mitosis. Second, the actin seems sensitive to external forces as it does not equally associate with all retraction fibres but instead concentrates at retraction fibres that are unequally distributed and therefore under most force. This was demonstrated by comparing the subcortical actin in cells dividing on bar- and disc-shaped micropatterns. On bars, actin concentrates at the retraction fibres at either end of the cell, whilst on discs – where retraction fibres are equally distributed around the cell and there is little/negligible anisotropy in force – the actin continues to circulate and does not show a polarized localization. Finally, the actin structures seem to help align the spindle by exerting pulling forces on astral microtubules: spindles were observed to move predominantly towards the subcortical actin and this movement was lost when astral microtubules were disrupted with nocodazole [14]. The next step in exploring these actin structures will be to specifically disrupt their assembly and investigate the affect on spindle orientation according to force, this may prove challenging as any disruption of these structures may also alter cortical actin.

Alongside actin, there is also evidence that the actin motor myosin-2 is involved in linking the spindle to mechanical forces [15], [25]. This makes sense, since in other contexts actin and myosin-2 are known to form contractile actomyosin networks that control the shape and mechanics of a cell and are in turn sensitive to the external mechanical environment [51], [52], [53]. In zebrafish embryos, treatment with the myosin-2 inhibitor blebbistatin disrupts the usual alignment of mitotic spindles with tensile forces that is seen during epiboly. However, treatment with blebbistatin also affected cell shape in the embryo – reducing the cell elongation normally seen along the axis of tension. The reduction in cell elongation was inevitably followed by a decline in the number of cells dividing along their longest axis and therefore along the axis of tension. However, further analysis showed that this decline was greater than would be expected if the cells were dividing according to shape alone, suggesting that myosin-2 may, in fact, be directly influencing spindle orientation, as well as controlling cell shape [15].

The story may be similar in cells at the periphery of the *Drosophila* wing imaginal disc, where myosin-2 shows a polarized distribution. These cells are under the greatest tensile stretch and have been shown to divide according to the axis of this stretch [25]. However, cells at the centre of the disc have a different mechanism for orienting their divisions, which involves another myosin, an unconventional myosin called Dachs. Dachs is localized in a planar polarized manner across the wing disc, localizing at the distal sides of each cell's apical surface [24], [54]. This localization is essential for regulating the proximal-distal orientation of cell division seen in the centre of the disc. A loss of Dachs, or an abnormal localization of Dachs around the entire cell, cause cell division orientation in the centre of the disc to become more random [24]. These cells also lose their normal proximal-distal elongated shape. In contrast, cells at the periphery of the disc do not require Dachs for their oriented divisions, with divisions actually occurring at an angle perpendicular to Dachs localization [23], [25]. Cells at the periphery are instead responding to anisotropic tissue tension and localizing myosin-2 to assemble actomyosin cables in a polarized manner [23], [25].

How does Dachs function in cells in the centre of the disc to determine cell division orientation? The answer appears to be that this myosin creates an internal anisotropy by altering cell shape. In keeping with this idea, Mao et al. observed a strong correlation between apical cell shape and division angle in the wing disc, with cells dividing along their long axis. Moreover, in the absence of Dachs, cells in the disc lost their elongated shape and had an increased apical area [24]. Based on these observations and mathematical models, Mao et al. proposed that Dachs regulates cell shape by exerting a contractile force on apical cell junctions, which constricts cell–cell junctions at the distal end of the cell (and ergo the proximal end of the neighbouring cell) resulting in cell elongation along the proximal-distal axis. Cells then follow the long axis rule to divide in a proximal-distal direction.

## 10 NuMA et al. may provide a direct link to the spindle

The evidence from single cells and tissues indicates that changes in the organization and localization of actin and myosins help translate changes in mechanical force to the mitotic spindle. But, what molecules provide the link between the spindle, the cell cortex and actin in this case? A strong possibility is that the same core molecules involved in orienting the spindle according to cell polarity are also involved in orienting the spindle according to mechanical force. These include NuMA, LGN, Gα and dynein/dynactin and while their role in standard spindle orientation has been extensively studied and reviewed [6], [55], their importance in orienting according to force is still unclear. However, a recent study in which cultured keratinocytes were exposed to a unidirectional stretch indicates that NuMA, at least, is involved [19]. Seldin et al*.* found that when wild type keratinocytes were stretched, spindle orientation robustly followed the axis of stretch. However, this alignment was lost in cells in which NuMA was knocked down. Intriguingly, while expression of full length NuMA in the knockdown cells rescued the spindle orientation according to stretch, a mutant form of NuMA, which lacked a domain for binding to the 4.1 family of proteins, did not [19]. The 4.1 family of proteins are known actin interactors, and function to help link the actin cytoskeleton to the cell cortex [56]. It is therefore tempting to speculate that NuMA might provide a direct link between the spindle and the actin cytoskeleton when mechanical force is applied.

## 11 Concluding remarks

Recent work in cultured cells and intact tissues has demonstrated that the mitotic spindle can orient according to external mechanical cues. However, this field is still in its infancy and there is much left to understand. In particular, our knowledge of the molecular mechanisms required to orient the spindle to external force is very sketchy. We know that actin and myosin are likely to be involved and that NuMA may provide a link between the spindle and changes in the localization/organization of actin. However, beyond that we have very little idea. For example, it will be of great interest to determine what sits upstream of actin organization in the transmission of external force to the spindle, one possibility is that known cellular “tension-sensors” such as vinculin and talin are involved [57], [58], [59]. We also still need to unravel the contribution of cell shape from a more direct mechanism linking force to the spindle; this is especially true in tissues where the two have yet to be conclusively separated. Finally, it is likely that the orientation of the mitotic spindle to mechanical cues is a widely used mechanism for coordinating cell division across complex tissues but, as yet, this has only been studied in the context of tissue morphogenesis and thus our understanding is very limited. It is tempting to speculate that linking force with spindle orientation could be a crucial mechanism in other tissue contexts where cell division is combined with a changing mechanical tissue environment, such as tumorigenesis, wound healing and the stem cell niche. It remains to be seen how mechanical forces might influence mitotic spindle orientation in these and other tissue contexts.
